# Two Distinct Integrin-Mediated Mechanisms Contribute to Apical Lumen Formation in Epithelial Cells

**DOI:** 10.1371/journal.pone.0019453

**Published:** 2011-05-06

**Authors:** Satu Marja Myllymäki, Terhi Piritta Teräväinen, Aki Manninen

**Affiliations:** Biocenter Oulu, Oulu Center for Cell-Matrix Research, Department of Medical Biochemistry and Molecular Biology, University of Oulu, Oulu, Finland; Medical College of Georgia, United States of America

## Abstract

**Background:**

Formation of apical compartments underlies the morphogenesis of most epithelial organs during development. The extracellular matrix (ECM), particularly the basement membrane (BM), plays an important role in orienting the apico-basal polarity and thereby the positioning of apical lumens. Integrins have been recognized as essential mediators of matrix-derived polarity signals. The importance of β1-integrins in epithelial polarization is well established but the significance of the accompanying α-subunits have not been analyzed in detail.

**Principal Findings:**

Here we demonstrate that two distinct integrin-dependent pathways regulate formation of apical lumens to ensure robust apical membrane biogenesis under different microenvironmental conditions; 1) α2β1- and α6β4-integrins were required to establish a basal cue that depends on Rac1-activity and guides apico-basal cell polarization. 2) α3β1-integrins were implicated in positioning of mitotic spindles in cysts, a process that is essential for Cdc42-driven epithelial hollowing.

**Significance:**

Identification of the separate processes driven by particular integrin receptors clarifies the functional hierarchies between the different integrins co-expressed in epithelial cells and provides valuable insight into the complexity of cell-ECM interactions thereby guiding future studies addressing the molecular basis of epithelial morphogenesis during development and disease.

## Introduction

The key property of epithelial cells, which line the surfaces and cavities throughout the body, is their ability to form two distinct surface domains, apical domain facing the outside environment and basolateral domain contacting the ECM and neighboring cells. Epithelial morphogenesis during development defines organ architecture by forming different types of tubes and glands with apical lumens. These apical compartments can form via multiple mechanisms [1], [2]. Cavitation involves clearance of selected cells in a cell cluster by means of apoptosis although other mechanisms such as autophagy may play an additional role [3]–[5]. Loss of matrix anchorage (anoikis) is thought to be the main trigger but secreted death factors may also contribute to lumenal cell death [6]. Hollowing of individual cells (cell hollowing) or clusters of cells (cord hollowing) is driven by polarized membrane trafficking machinery and orientation of cellular cytoskeleton according to extracellular cues [1], [2], [7]. Cues from the extracellular microenvironment not only direct the positioning of the forming apical lumen but also govern the mechanism by which it is formed [5], [8], [9].

β1-integrins, which function as αβ-heterodimers, are important ECM-receptors implicated in conveying the polarity cues from the ECM [9]. However, the contributions of specific integrin heterodimers in these processes have not been addressed in detail. In this study we have analyzed the specific roles of different integrin heterodimers in the formation of apical membrane using 3D cultures of Madin Darby Canine Kidney (MDCK) epithelial cells. It was found that two distinct integrin-dependent pathways regulate epithelial cystogenesis. Whereas α2β1- and α6β4-integrins were required for apical lumen formation in collagen gels, α3β1-integrin function was critical in BM-extract (BME) gels. Importantly, despite being mechanistically distinct, these integrin-dependent pathways were found to complement each other functionally to ensure efficient cystogenesis under different ECM environments.

## Results

### Characterization of the adhesive properties of integrin-KD MDCK cells

**Figure 1:**
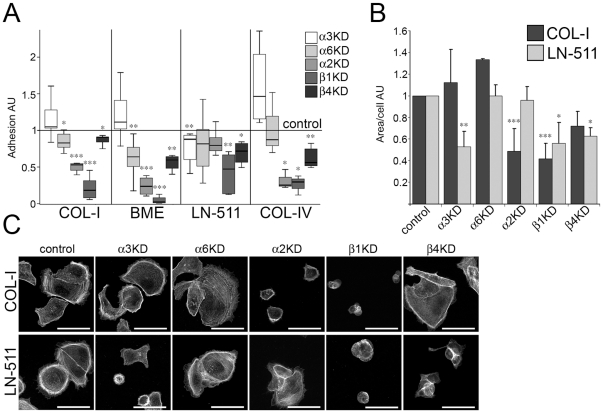
Adhesive properties of the integrin-depleted MDCK cells. A) Single cell suspensions of control, Itgα3-, Itgα6-, Itgα2-, Itgβ1- and Itgβ4-KD MDCK cells were allowed to settle for 90 minutes on collagen I (COL-I)-, basement membrane-extract (BME)-, laminin-511 (LN-511)- or collagen IV (COL-IV)-coated tissue culture wells. Non-adherent cells were washed away and remaining adherent cells were fixed, stained and quantified. Adhesion of control cells to each coating was set to 1 and adhesion of the different Itg-KD cells is shown relative to control (arbitrary units, AU). Each Itg-KD sample represents data from 3–10 independent experiments and each value is normalized to a control value within the experiment. The spread of normalized values from the lowest to the highest, together with the 25^th^, 50^th^ (median) and 75^th^ percentiles is shown. P-values <0.05 are signified by (*), <0.01 by (**) and ≤0.001 by (***). B) Control, Itgα3-, Itgα6-, Itgα2-, Itgβ1- and Itgβ4-KD MDCK cells were plated on COL-I or LN-511-coated glass coverslips and allowed to spread for 90 minutes. Cells were fixed and filamentous actin was stained using TRITC-Phalloidin. C) Quantitation of the cell spreading data on collagen I (upper panel) and on LN-511 (lower panel). The combined data shows the mean +SD of 2–6 independent experiments. For each experiment and coating condition, ∼81–250 cells from 11 pictures were analyzed per one sample. Data is shown as KD/control ratio (Arbitrary units, AU) of the spreading area per cell due to significant variation between coating efficiencies in separate experiments. P-values <0.05 are signified by (*), <0.01 by (**) and P-values ≤0.001 by (***).

The expression profile of different integrins in MDCK cells was studied using a quantitative PCR (qPCR) analysis that revealed abundant expression of several integrin chains, including β1-, β3-, β4-, β5- β6-, β8, α2-, α3-, α6- and αV-subunits (Figure S1A). Integrin mRNA expression levels were determined in three different culture conditions used in this study; 1) subconfluent on tissue culture plastic, 2) cells grown for 6 days in 3D collagen I gels and 3) 3D cultures in BME gels grown for 3 days. Notable reduction in mRNA levels was observed for β1- and α2-subunits seeded into BME gels and for α6- and β1-subunits embedded into collagen when compared with 2D cultures indicating that cellular microenvironment controls integrin expression. To address the functional roles of the most abundant laminin- (α3β1, α6β1, α6β4) and collagen-binding (α2β1) integrins we generated retroviral shRNA-knockdown (KD) constructs targeting α2-, α3-, α6-, β1 and β4-subunits. Efficient depletion of the specific target mRNAs was confirmed by qPCR (Table S1). Down-regulation of protein levels was demonstrated either by western blotting or by immunofluorescence (Figure S1B). Adhesive properties of the integrin-KD (Itg-KD) MDCK cells were characterized by employing a standard adhesion assay on selected substrates. Itgβ1-KD cells lacking functional β1-integrin heterodimers showed marked adhesion defects on all substrates (Figure 1A). Inhibition of the Itgβ4- or individual Itgα-subunits revealed more modest and/or specific defects. All of these KDs slightly reduced adhesion on LN-511 agreeing with the reported redundancy between different laminin-receptors in MDCK cells [10]. Depletion of Itgα2-subunit, component of the collagen receptor α2β1, had prominent effects on adhesion to collagens and BME, suggesting that adhesion to laminin-rich BME was mainly mediated via α2β1-integrin/collagen IV interactions. Depletion of α6- or β4-subunits of the α6β4-integrins diminished adhesion to BME and collagen IV to some extent. Itga3-KD cells had a tendency to adhere better on collagens and BME, which may reflect its proposed role as a negative regulator of other integrins [11]. However, these positive effects observed in Itgα3-KD cells were not statistically significant.

To analyze the specific integrin-ECM interactions in more detail, we studied cell spreading by seeding Itg-KD cells on collagen I or laminin-511 coated surfaces and compared average cell areas (Figure 1B and C). Itgβ1- and Itgα2-KD cells spread poorly on collagen I indicating that α2β1-integrin is crucial not only for adhesion but also for cytoskeletal reorganization upon binding to collagen. Whereas individual Itgα-subunits did not markedly reduce adhesion to LN-511, Itgα3- and β1-subunits were required for efficient cell spreading on laminin. Curiously, whereas Itgα6-KD cells displayed no spreading defects, depletion of the β4-subunit inhibited spreading on LN-511. Why depletion of α6- or β4-integrins led to differing phenotypes is not clear but it has been reported that the cytoplasmic tail of β4-integrin possesses some signaling capacity [12] and is sufficient for α6-subunit-independent recruitment of hemidesmosomal components [13]. The specificities of the RNAi phenotypes were confirmed by using independent shRNA constructs (Figure S2A and B). This data indicated that cytoskeletal reorganization upon binding to collagen I and LN-511 were regulated by α2β1- and α3β1-integrins, respectively. In addition, β4-integrins contributed to cell spreading on LN-511.

### Integrins play distinct roles in epithelial cystogenesis

**Figure 2:**
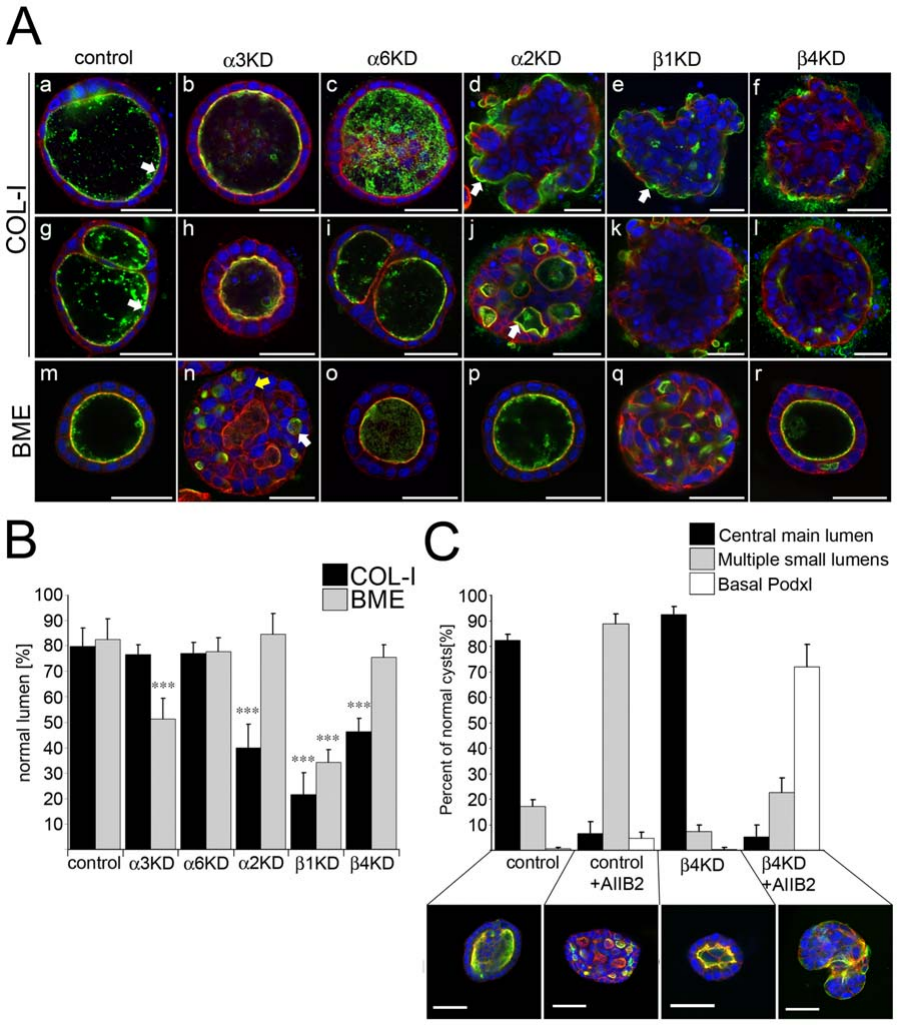
Epithelial cystogenesis in collagen requires α2β1- and β4-integrins but depends on α3β1-integrin in BME gels. A) Control, Itgα3-, Itgα6-, Itgα2-, Itgβ1- and Itgβ4-KD MDCK cells were grown in 3D collagen I matrix for 10 days (Upper panels, COL-I) or in 3D BME gel for 7 days (lower panel, BME). Cysts were fixed and stained for DNA (DAPI, blue), filamentous actin (red) and an apical marker podocalyxin (green). Cysts were phenotypically classified as normal when they had 1–2 central main apical lumen(s) with smooth contour. Cysts with poorly organized lumens, multiple small lumens or with no lumen were scored as abnormal. B) Quantitation of the cyst phenotypes in collagen I and BME. The data shows averages +SD from 3–6 independent experiments. A minimum of 150 cysts per sample was scored in each experiment. P-values <0.01 are signified by (**) and P-values ≤0.001 by (***). C) BME-embedded control and Itgβ4-KD cysts were grown in the presence or absence of 5 µg/ml Itgβ1-function-blocking antibody (AIIB2) for 7 days. Cysts were stained as in A) and phenotypically classified into three categories; 1) normal (central main lumen), 2) multiple small lumens and 3) basal localization of Podxl. Data shows averages +SD from two independent experiments with duplicate samples. A minimum of 100 cysts were analyzed per sample. Size bars are 30 µm.

To study the role of specific integrins in the biogenesis of apical lumen we embedded control cells and the different Itg-KD cells into 3D collagen I gels where parental MDCK cells form polarized epithelial cysts [14]. When cultured in collagen I gels for 10 days, the majority (∼80%) of control cells formed cysts with dilated central lumens (Figure 2A). Cysts with smooth-surfaced circular lumen(s) stained with apical marker podocalyxin (Podxl), whose subcellular localization is known to be restricted to free surfaces [15], were classified as normal (arrows in Figure 2Aa and Ag). Unlike the control cysts, the majority of Itgβ1- and Itgα2-KD cysts lacked big central lumens and instead grew as poorly organized clusters of cells with uneven basal surface (Figure 2Ad, Ae, and Ak). Approximately half of the abnormal Itgα2-KD cysts contained small lumens that accumulated apical markers indicating some degree of cell polarity (arrow in Figure 2Aj). However, many Itgα2-KD and most Itgβ1-KD cysts appeared unpolarized and mistargeted apical pole marker Podxl to the basal plasma membrane facing the ECM (arrows in Figure 2Ad and Ae). This finding is in line with an earlier report where a function-blocking antibody against integrin α2-subunit inhibited tubulocyst formation upon collagen overlay [16]. Similar to spreading data, Itgβ4- and Itgα6-KD cells behaved differently as the former showed a phenotype similar to Itgα2- and Itgβ1-KD cells (Figure 2Af and Al). Itgα6-KD cells, on the other hand, formed polarized cysts with essentially normal morphology. Itgα3-KD cysts contained central lumens and were properly polarized although it was noted that Itgα3-KD cells had a slightly elevated susceptibility to apoptosis and grew more slowly than controls upon seeding to collagen gels. Also 2D cultures of Itgα3-KD cells were found to proliferate with slower kinetics and to contain apoptotic cells more frequently than the controls (data not shown).

Mostov and coworkers demonstrated that addition of exogenous laminin at least partially bypasses the requirement of β1-integrin activation for cystogenesis [8]. To examine if exogenous laminin can revert the observed cystogenesis phenotypes, all the integrin-KD MDCK cells were embedded into 3D BME-gels rich in laminin. Whereas the cystogenesis defects of Itgα2- and Itgβ4-KD cells appeared to be completely rescued, Itgβ1-KD cysts were still grossly abnormal and contained either no lumen or many small lumens (Figure 2Aq). In both cases, however, Podxl staining disappeared from the basal membrane (compare Figures 2Ae and 2Aq). Given the laminin spreading and collagen cystogenesis phenotypes of Itgβ1- and Itgβ4-KD cells we next addressed the possibility that β4-integrin may partially rescue the polarity by conveying the basal signal in BME environment where high concentration of laminin is provided basally. To this end, we studied the localization of Podxl in control and Itgβ4-KD cells in BME gels treated or not with β1-function-blocking antibody (AIIB2). Whereas β1-blocking antibody-treatment in control cells resulted in multiple lumen phenotype similar to Itgβ1-KD cells, combining AIIB2-treatment with Itgβ4-KD led to a severe polarity defect similar to Itgβ1-depleted cells in collagen (Figure 2C). Apical markers were retained basally in Itgβ4-KD/Itgβ1-inhibited cells suggesting that β4-integrins can mediate a basal cue in Itgβ1-depleted cysts in BME gels.

Interestingly, significant number of Itgα3-KD cysts, which were seemingly normal in collagen gels, failed to form a central lumen and instead displayed multiple small rudimentary lumens (white arrow in Figure 2An). When compared with cells in the Itgβ1-KD cysts, individual Itgα3-KD cells had more cuboidal morphology and were seemingly polarized and connected to small apical compartments in the cysts. However, the overall cellular organization was abnormal. While a single layer of cells surrounded the lumens in control cysts, Itgα3-KD cysts frequently contained multiple cell layers (yellow arrow in Figure 2An). Itgα6-KD cells showed no overt cystogenesis phenotype in BME-gels. Experiments with alternative shRNA constructs yielded similar results (Figure S2C and D). These experiments suggest that different integrin-mediated cascades orchestrate efficient cystogenesis, α2β1- and α6β4-integrins seem to regulate establishment of apico-basal polarity whereas α3β1-integrins contribute to cystogenesis in BME environment by an unknown mechanism.

### α2β1- and α3β1-mediated mechanisms to form apical lumens are mechanistically distinct

A recent study by Martin-Belmonte *et al.* reported that polarization dynamics, depending on the strength of the polarity cue and the initial size of unpolarized cell clusters, both apoptotic and non-apoptotic mechanisms contribute to cystogenesis in MDCK cells [5]. However, it remains unclear whether different molecular machineries at the cell/ECM-interface are involved in such regulation. Because Itgα2-KD cysts were normal in BME gels and Itgα3-KD cysts were normal in collagen gels it is likely that these two integrins are not in a linear pathway, but rather seem to constitute two independent or parallel mechanisms.

**Figure 3:**
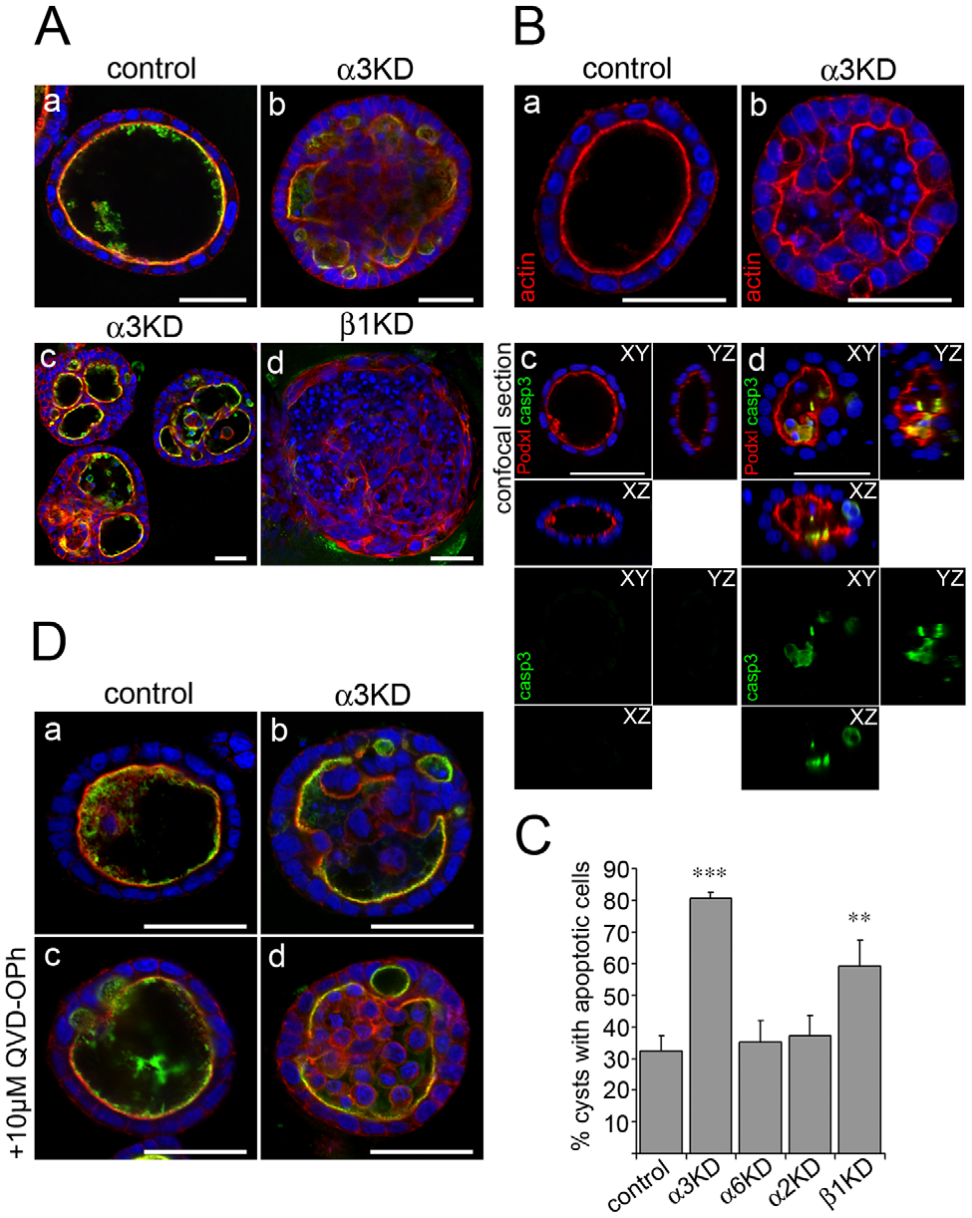
BME-embedded Itgα3-KD MDCK cysts can form lumens via apoptotic cavitation. A) Control, Itgα3- and Itgβ1-KD MDCK cells were grown in 3D BME-gels for 15 days. Cysts were fixed and stained for filamentous actin (TRITC-phalloidin, red), an apical marker (podocalyxin (Podxl), green) and nuclei (DAPI, blue). B) Upper panel: Control and Itgα3-KD MDCK cells were grown in 3D BME-gels for 7 days. Cysts were fixed and filamentous actin was stained using TRITC-phalloidin (red). Lower panel: Cysts were fixed and stained for an apical marker podocalyxin (Podxl, red) and for an apoptosis marker cleaved caspase-3 (green). In both panels nuclei were stained using DAPI (blue). C) Quantitation of cysts with apoptotic cells in the lumen. Control, Itgα3- Itgα6-, Itgα2- and Itgβ1-KD MDCK cells were grown in 3D BME gels for 7 days, fixed and stained for filamentous actin and nuclei as mentioned above. The data is shown as average +SD and is representative of 2–3 independent experiments. P-values <0.01 are signified by (**) and ≤0.001 by (***). D) Control and Itgα3-KD cells were grown in 3D BME gels for 7 days in the presence or absence of 10 mM apoptosis inhibitor QVD-OPh. Cells were fixed and stained for podocalyxin (Podxl, green), filamentous actin (red) and DNA (blue). Size bars are 30 µm.

Upon prolonged culture (followed up to 15 days) in BME gel it was noted that the small apical lumens in Itgα3-KD cysts grew in size and occasionally seemed to fuse to form a more central lumen (Figure 3Ab and c). These lumens contained a significant number of small nuclei with no detectable actin staining indicating cell borders thus suggesting that these may represent apoptotic cells (arrow in Figure 3Bb). In addition to nuclear morphology, apoptosis was determined by staining the cysts for cleaved caspase-3 (Figure 3Bc and d). To analyze the involvement of apoptotic cavitation in the cystogenesis in BME gels in more detail we screened for apoptotic nuclei in the different integrin-KD cysts. It was found that while the control cells typically formed lumens via non-apoptotic mechanisms, Itgα3- and, to lesser extent, Itgβ1-KD cysts contained significant amount of lumenal apoptosis (Figure 3C). Itgα2-KD cysts did not show lumenal accumulation of fragmented nuclei suggesting that lumens formed via hollowing. Both apoptotic and non-apoptotic mechanisms seemed to be inhibited in BME-embedded Itgβ1-KD cysts in which apical lumens did not grow in size upon prolonged culture (Figure 3Ad). When QVD-O-Ph, an apoptosis inhibitor, was added to cyst cultures, a robust accumulation of lumenal cells was observed in Itgα3-KD cysts whereas very few were seen in control or Itgα2-KD cysts that continued to develop normally (Figure 3D and data not shown).

This data indicated that non-apoptotic (hollowing) lumen formation is abrogated in BME gel-embedded Itgα3-KD cysts but lumens can still form via apoptotic cavitation. Taken together these findings suggest that although β1-integrins are important for efficient lumen formation under all conditions tested, α2β1- and α3β1-integrins operate in two distinct pathways. Next, we went on to characterize these affected pathways in more detail.

### α2β1-integrin mediated pathway is required for the establishment of a basal cue that guide apico-basal polarization

**Figure 4:**
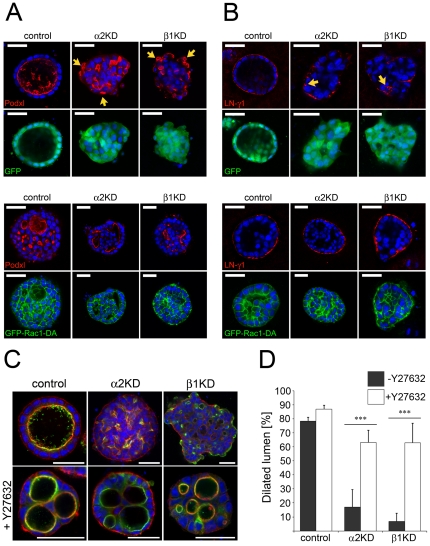
Defective basal cue in α2β1-integrin-depleted cysts is rescued by Rac1 activation or inhibition of RhoA. A) Control, Itgα2- and Itgβ1-KD MDCK cells were transduced with a GFP- (upper panels) or Rac1^G12V^-GFP-expressing (lower panels) retroviruses. Transduced cells were seeded into 3D collagen gels and grown for 10 days to allow formation of cysts. Cysts were fixed and stained for A) an apical membrane marker podocalyxin (Podxl) or B) for laminin-γ1 (LN-γ1, red, left panels). Grey lines in B depict the basal surface of the cysts as defined by the GFP-fluorescence. Yellow arrows depict mislocalized basal Podxl staining in A) and abnormal fragmented laminin-deposits in B) observed in the Itgα2- and Itgβ1-KD cysts. Nuclei were visualized using DAPI (blue). Size bars are 30 µm. C) Control, Itgα2- and Itgα1-KD cells were grown in 3D collagen gel for 10 days in the presence or absence of 10 µM Y27632. Cysts were fixed and stained as above. Cysts were phenotypically classified as normal when they had major dilated apical lumen(s) with smooth contour. Cysts with poorly organized lumens, multiple small lumens or with no lumen were scored as abnormal. D) The cyst phenotypes were quantified. The data shown includes averages from 3–4 independent experiments. A minimum of 200 cysts per sample was scored in each experiment. P-values ≤0.001 are signified by (***). Size bars are 30 µm.

β1-integrin-dependent assembly of laminin guides cell polarization in collagen-grown MDCK cysts [8]. Thus, abnormal laminin assembly may underlie the cystogenesis defect in Itgα2-, Itgβ1- and Itgβ4-KD cysts. In some cases laminin deposits were observed seemingly laterally between the cells (see arrowheads in Figure 4B). However, the fact that the overall morphology of the affected Itg-KD cysts was disturbed complicates the assessment of the integrity of their BME. Because laminin was still secreted and at least to some extent assembled in α2β1- and (α6)β4-integrin-depleted cysts, it is possible that the seemingly abnormal BME staining in Itgα2-, Itgβ1- (Figure 4B) and Itgβ4-KD (data not shown) cells merely reflect their perturbed cellular organization. On the other hand, basal staining of the free surface marker Podxl observed in these Itg-KDs (Figure 2A) strongly supports a conclusion that the establishment of ECM-triggered basal cue is affected. A small GTPase Rac1 reportedly mediates this basal cue and it was reported that forced activation of Rac1 bypasses the requirement of β1-integrin activation [9]. We measured the steady-state levels of active Rac1 in the control and the different Itg-KD MDCK cysts. However, basal Rac1 activity was low and no Itg-subunit specific effects were seen as all the Itg-KDs tended to have slightly reduced levels of active Rac1 (data not shown).

To overcome this problem we first addressed the functional role of Rac1-activation by utilizing a cell line in which expression of dominant negative Rac1 can be regulated by doxycycline (T23 MDCK-Rac1^T17N^-Tet-Off, [17], Figure S3C). T23 MDCK-Rac1^T17N^ cells were seeded into the different 3D gels in the presence or absence of doxycycline. We could confirm the role of Rac1 as normal cysts formed in the presence of doxycycline, but induced expression of Rac^T17N^ led to appearance of non-polarized cell clusters in both collagen I and BME environments (Figure S3A and B) resembling the phenotypes of Itgα2-, Itgβ1- and Itgβ4-KD cysts in collagen (Figure 2). The possibility that these cyst phenotypes resulted from impaired Rac1-activation was further assessed by overexpressing a dominant-active GFP-tagged form of Rac1 (GFP-Rac1^G12V^) in Itgα2- and Itgβ1-KD cells grown in collagen. Constitutive Rac1-activation smoothened the contour of basal surfaces, led to more continuous basal laminin network (Figure 4B) and, importantly, exclusion of an apical pole marker Podxl from the cell-matrix contact sites (arrows in Figure 4A). Although the ECM-contacting cell surfaces adopted basal identity in these rescued Itg-KD cysts, apical lumens did not form properly. This is probably due to effects of aberrant Rac1-activation on survival or motility of luminal cells as GFP-Rac1^G12V^ perturbed lumen biogenesis in a similar manner also in control MDCK cysts (Figure 4A and B). Others have also reported similar findings on the effects of Rac1^G12V^ on cystogenesis [18]. Rac1-activation plays multiple roles during epithelial morphogenesis and it is crucial for not only for the initial signal guiding basal laminin assembly in collagen gels but also for the laminin-dependent polarity cue that orients apico-basal cell polarity in BME environment.

One of the well-documented consequences of Rac1-activation is its reciprocal regulation of the RhoA/ROCK/MyosinII-pathway [19]. Indeed, recent data has implicated RhoA inhibition in the establishment of the basal cue in MDCK cysts [20]. Yu and coworkers showed that the polarity defect in cysts where integrin signaling was blocked could be rescued by inhibiting the RhoA/ROCK1/MyosinII-pathway. To assess if the phenotype observed in our model system could be similarly rescued, we treated collagen-embedded control, Itgα2- and Itgβ1-KD cysts with a ROCK-inhibitor Y27632. Control cysts remained properly polarized in the presence of Y27632 although they often formed 2–3 smooth-surfaced lumens instead of one main lumen (Figure 4C). Why Y27632 led to formation of multiple big lumens is unclear. Importantly, the cell polarity was rescued in most of the Y27632-treated Itgα2- and Itgβ1-KD cysts that now displayed very similar overall morphology than the ROCK-inhibitor-treated control cysts (Figure 4C, D). This data suggests that the phenotype of α2β1-integrin-depleted cells is due to perturbed signaling activities of Rac1 and RhoA-GTPases. Taken together, our data strongly suggest that impaired activation of Rac1/RhoA-mediated signaling cascade that is essential for the establishment of basal cue underlies the cystogenesis defect in α2β1- and (α6)β4-integrin-depleted cells.

### α3β1-integrins are required for proper orientation of mitotic spindles in BME gels

Cdc42, another small GTPase of the Rho-family, is an important component of the cell polarity signaling machinery required for apical hollowing process [5], [21]. Cell division axis in polarized epithelial cells is tightly regulated and follows a defined sequence of events in both 2D and 3D culture systems [22], [23]. First, subapical centriole duplicates and one the centrioles migrates towards the basal side of the nucleus. Second, during early prophase the forming mitotic spindle aligns parallel to apico-basal axis but then rotates 90° such that the metaphase spindle is perpendicular to apico-basal axis. Interestingly, inhibition of Cdc42 function in epithelial 3D cultures leads to very similar multilumen phenotype as was observed here for Itgα3-KD cells [24], [25]. Hall and coworkers provided evidence that the multilumen phenotype in Cdc42-depleted cysts is due to misaligned spindles in dividing cells of the growing cysts [23], [25].

**Figure 5:**
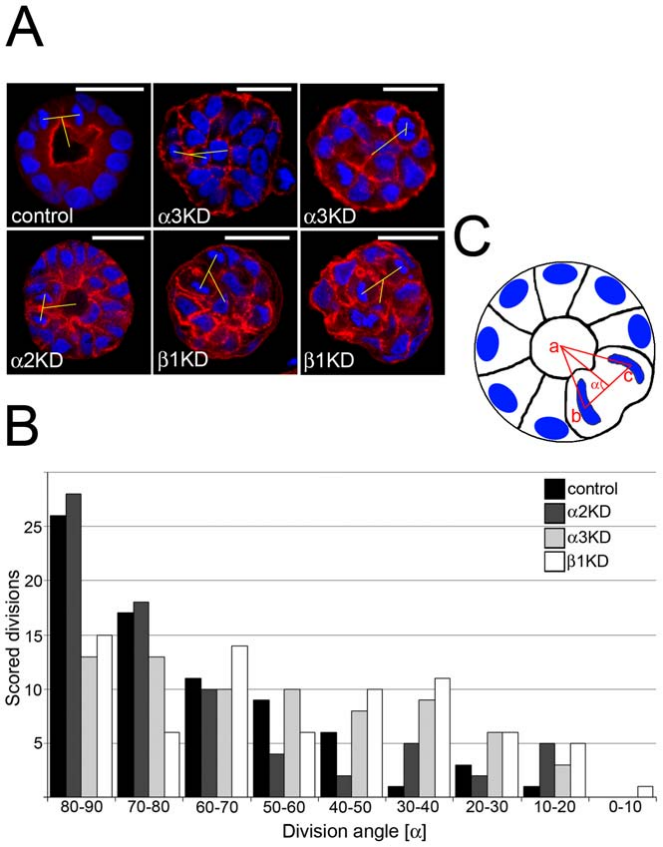
α3β1-integrin is required for mitotic spindle orientation. A) Control, Itgα3-, Itgα2- and Itgβ1-KD MDCK cells were grown in 3D BME gel for 4 days, fixed and stained for nuclei (DAPI) and actin (TRITC-phalloidin). Confocal XY-sections from the middle of the cysts are shown. Sizebar is 30 µm. B) To analyze the orientation of cell division axis in cysts, stacks containing 74 mitotic post-metaphase cells from control, Itgα3-, Itgα2- and Itgβ1-KD cysts were collected. A frequency distribution of KD and control cells according to division angle is shown (P≤0.001 for Itgα3- and β1-KD). C) The XYZ-coordinates of cyst centre (a) and the segregated chromosomes of the dividing cell (b & c) were measured as shown in the schematic. The division angles (α) were calculated as described in materials and methods.

To characterize the observed multilumen phenotype of Itgα3-KD cells in BME gels in more detail, control, Itgα3-, Itgα2- and Itgβ1-KD cysts were grown for 4 days after which dividing cells were visualized by staining nuclei and actin. The angles of division in relation to the center of mass of the forming cysts were determined. To avoid possible error resulting from rotating metaphase plates, only post-metaphase cells (including mitotic cells between anaphase to late telophase/cytokinesis) were analyzed in which the coordinates of the separated post-metaphase chromatids relative to cyst center could be determined. The majority of the dividing cells in control and Itgα2-KD cysts divided perpendicular to the forming apico-basal axis (Figure 5A). In contrast, many Itgα3-KD and Itgβ1-KD cells divided at sharper angles relative to cyst center. The distribution of the angles of mitotic divisions in Itgα3- and Itgβ1-KD cysts was significantly broader than in controls suggesting that defective orientation of mitotic spindles could underlie the observed multilumen phenotypes (Figure 5B). Again the fact that Itgα3-KD cysts were in general less well organized than controls complicated the assessment how much the observed difference in the cell division axes contributes to the lumen formation. To follow cell divisions in the developing cysts in a more dynamic fashion, we set up a time-lapse assay. For this purpose we generated MDCK cells stably expressing mouse E-cadherin fused to GFP (ECad-GFP). The majority of Ecad-GFP-expressing cells formed polarized cysts with prominent lumens confirming that cystogenesis was not perturbed by the ectopically expressed Ecad-GFP or the imaging conditions used (data not shown). Importantly, after reaching a certain cluster size, cells in control cysts almost invariably seemed to divide in the plane of the epithelial monolayer surrounding the forming lumen (Figure S4A and movie S1). On the contrary, in Itgα3-KD cysts cell division axes had variable orientations and were difficult to determine even at later stages of cystogenesis (Figure S4B and movie S2).

**Figure 6:**
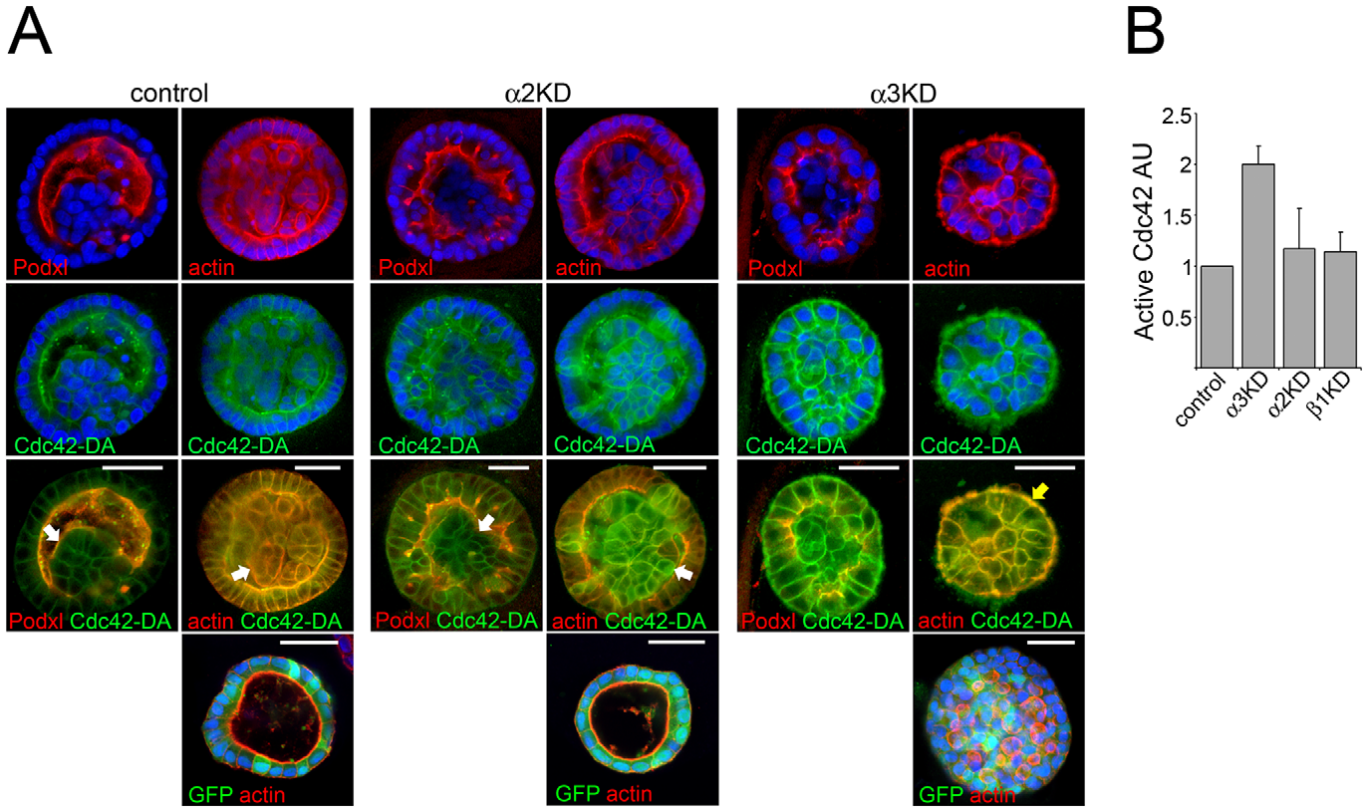
α3β1-integrin modulates the function of Cdc42. A) Control, Itgα2- and Itgα3-KD MDCK cells were transduced with GFP- or Cdc42^Q61L^-GFP expressing retroviruses. Transduced cells were seeded into 3D BME-gels and grown for 7 days to allow formation of cysts. Cysts were fixed and stained for actin (red, right panels) or podocalyxin (red, left panels). Active Cdc42-GFP is shown in green. Nuclei were visualized using DAPI (blue). B) Control, Itgα3-, Itgα2- and Itgβ1-KD MDCK cells were seeded onto BME-gel and grown to confluency. Cells were lysed and the amount of GTP-loaded Cdc42 in the lysates was measured using G-LISA™ assay. The data is shown as mean +SD and is representative of 2 independent experiments. Sizebars are 30 µm.

Given the central role of Cdc42 in the epithelial hollowing and the similarity of the Itgα3-KD cystogenesis phenotype with that reported for Cdc42-depleted cells we next addressed the possible involvement Cdc42 regulation in the different integrin-depleted MDCK cells. Cells were grown on top of BME gels and allowed to reach confluency prior to determination of Cdc42 activity. The steady state activity of Cdc42 was relatively low in all of the cell lines studied but Itgα3-KD cells displayed a modest two-fold increase suggesting that α3β1-integrin might contribute to negative regulation of Cdc42 (Figure 6B). The possible role of Cdc42 as a downstream effector in the α3β1-integrin-mediated pathway was further investigated by expressing a GFP-tagged dominant-active mutant of Cdc42 (GFP-Cdc42^Q61L^) in the control, Itgα2- and Itgα3-KD cells. Overexpression of GFP-Cdc42^Q61L^ elongated the lateral membrane domains resulting in columnar morphology in all cell types (Figure 6A). Whereas Cdc42 activation had no significant effects on the morphology of Itgα3-KD cysts which still contained either no or small lumens, it did perturb the cystogenesis of control and Itgα2-KD cells which often contained multiple cell layers and lumenal cell populations thereby resembling the phenotype of Itgα3-KD cysts (white arrows in Figure 6A). Interestingly, whereas in control and in Itgα2-KD cells GFP-Cdc42^Q61L^ localized mainly to lateral and apical membranes, basal accumulation of Cdc42 was observed in many Itgα3-KD cysts (Figure 6A, yellow arrow). This signal coincided with robust basal actin-staining typical for Itgα3-KD cysts thus raising the possibility that abnormal recruitment and/or activation of Cdc42 at the basal surface could be the underlying cause of the basal actin accumulation observed especially in cysts grown from selected Itgα3-KD cell clones with efficient silencing of Itgα3 mRNA. Although further studies are certainly needed to establish molecular connections between α3-integrin and Cdc42, these data suggest that α3β1-integrin may participate in the regulation of cellular localization and/or activity of Cdc42 during cystogenesis. Nevertheless, our data show that, similar to Cdc42-KD cells [25], Itgα3-KD cells fail to properly orient their mitotic spindles despite apparently normal apico-basal polarity. This defect disrupted cellular organization in the forming Itgα3-KD cysts and likely contributes to the observed multilumen phenotype.

## Discussion

Extracellular matrix is a dynamic scaffold that conveys environmental signals to regulate epithelial morphogenesis during organogenesis. Here we found that two separate integrin-mediated signaling cascades regulate formation of apical lumens in epithelial cells. Importantly, although these two pathways were mechanistically distinct, a cystogenesis defect in Itgα3-depleted cells was functionally alleviated by the α2β1/α6β4-cascade.

α2β1- and α6β4-integrins were found to be required to establish a Rac1-dependent basal cue upon binding to collagen matrix leading to apico-basal polarization of the ECM-contacting cell layer. Subsequently, cells entrapped in the forming lumen are cleared by apoptotic cavitation. Because depletion of the laminin-binding β4-subunit in collagen gels resulted in a phenotype comparable to those of Itgα2- or Itgβ1-KD cells, it appears that α2β1 (which is capable of interacting with both collagens and laminins) alone is not sufficient for generate a laminin-dependent basal cue that would then be conveyed by α6β4-integrins. Instead, it seems more likely that both α2β1 and α6β4 are required to establish the basal laminin-dependent cue while either one alone is sufficient to convey this signal once the cue is present. Interestingly, only depletion of β4-subunit resulted in cystogenesis phenotype whereas Itgα6-KD cells appeared largely normal despite robust reduction of both mRNA and protein levels of the α6-integrin subunit. It has been reported that (the cytoplasmic tail of) β4-integrin is sufficient for the recruitment of other hemidesmosomal components and has signaling capacity of its own [12], [26]. Although it is currently unclear if β4-subunit can reach the cell surface in the absence of α6-subunit, α6-subunit seems to be dispensable for both of these functions as well as for the cystogenesis phenotypes observed in this study.

Once BM is formed or if cells are embedded into laminin-rich BME, apical lumen is generated and maintained by hollowing that is in part regulated by Cdc42-regulated orientation of mitotic divisions [24], [25]. α3β1-integrins were found to be involved in guiding the orientation of cell division axis in the forming cysts in BME gels. Interestingly, the basal activity of Cdc42 seemed to be stimulated in Itgα3-KD cells. Moreover, a dominant active Cdc42^Q61L^ accumulated at basal side of the Itgα3-KD cysts whereas it was predominantly apical when expressed in control cysts. It is tempting to speculate that α3β1-integrins, for example by recruiting Cdc42-GTPase-activating proteins or some other factors function to restrict Cdc42-activity at the cell-ECM contact site and thereby contribute to the signal guiding the orientation of mitotic spindles. On the other hand, previous work has shown that phosphatidylinositol 3,4,5-trisphosphate PI(3,4,5)P_3_ is restricted to basolateral membrane domain while PI(4,5)P_2_ accumulates at the apical membrane where it seems to contribute to apical targeting of Cdc42 [24], [27]. It is possible that integrin-mediated signaling could regulate, at least to some extent, generation and/or maintenance of specific phosphoinositide domains and thereby affect localization of the key down-stream effectors, such as Cdc42. However, direct mechanistic links and the putative integrin-species specific regulatory components remain to be determined in future studies. In addition to spindle orientation, Cdc42-activation plays a role in the regulation of biosynthetic basolateral membrane trafficking, a process that could potentially contribute to the observed hollowing defect in Itgα3-KD cysts [28]–[30]. However, we have not observed significant biosynthetic membrane trafficking phenotypes in Itgα3-KD cells (unpublished data) suggesting that distinct Cdc42-signaling complexes participate in the two processes and only the former is regulated by α3β1-integrins.

The seemingly differing roles of α2β1/α6β4- and α3β1-integrins in the regulation of Rac1-, RhoA- and Cdc42-mediated pathways during cystogenesis is an interesting finding. RhoGTPases are crucial regulators cytoskeletal dynamics, epithelial morphogenesis and differentiation [31]. More than 20 RhoGTPase genes have been described in mammals and they are accompanied by a daunting number of regulatory proteins. The most studied Rho-family members, Rac1, Cdc42 and RhoA, all regulate integrin signaling but via somewhat different mechanisms [32]–[34]. The functions of the different RhoGTPases in 3D epithelial culture systems are also clearly distinct [8], [18], [20], [24], [35]. Integrin-mediated adhesion activates both Rac1 and Cdc42 but their activation profiles differ due to complex network of regulatory feedback loops that are incompletely understood [31], [36]. The role of specific integrins in determining the activation profiles of particular RhoGTPases has not been systematically addressed although different roles for α2β1- and α3β1-integrins in the regulation of RhoA-activation have been reported [37], [38]. We are not aware of studies systematically addressing the potential integrin-mediated spatial regulation of specific RhoGTPases. Although more detailed analyses are certainly needed, this current study suggests that α2β1/α6β4-integrins function to regulate Rac1/RhoA-dependent basal cue whereas α3β1-integrins seem to contribute to Cdc42-dependent positioning of mitotic spindles. As discussed above, such functional specificity could result from selective recruitment of effector complexes to different integrin adhesions. One indication to this direction could be our observation that although α3β1 also contributed to basal Rac1-activity on collagen (data not shown), it could not rescue the basal cue in Itgα2-KD cells. Interestingly, recent studies implicated two distinct Cdc42-specific GEFs, Intersectin-2 and Tuba, as regulators of mitotic spindle orientation [39], [40]. Intersectin-2 localized to centrosomes whereas Tuba was found subapically. Although both Intersectin-2 and Tuba regulated Cdc42-activity, spindle defects in Tuba-deficient cells could not be rescued by overexpression of Intersectin-2 suggesting that they activate Cdc42 in distinct complexes [39]. Clearly, detailed further studies are warranted to address the possible mechanisms how specific integrins might direct RhoGTPase activation to different cellular locations.

Further complexity arises from the fact that interacting effectors and co-receptors themselves largely define the function of integrin signaling. For example, α3β1-integrin appears to participate in two distinct signaling complexes, one at the cell-ECM contact sites and another at adherens junctions where it regulates E-cadherin function and the stability of cell-cell contacts [41]–[43]. A lot of interaction data exists on specific sets of cytoplasmic effectors that associate with different integrin β-subunits but less is known about integrin α-subunits [44]. Which are the molecules that could determine the specificity of, for example, α3β1- and α2β1-integrin-mediated signals in the regulation of cystogenesis is an important question for further studies.

The current findings again highlight the fact that integrins are much more than mere adhesion receptors and reveal a complex functional interplay between different integrins that is not limited by their ligand specificity. Having two redundant mechanisms that depend on different ECM signals is likely to be more error-proof than two pathways triggered by a common cue. Given the importance of robust apical membrane biogenesis during generation and maintenance of epithelial tissues, such plasticity is likely to be crucial to ensure robust epithelial polarization and morphogenesis *in vivo*.

## Materials and Methods

### Antibodies and Reagents

Mouse [45] and rabbit anti-podocalyxin (Podxl, gp135) were a kind gift from Dr. K. Simons and mouse anti-myc (9E10) epitope antibody was from Dr. D. Dreschler (both from Max-Planck Institute of Molecular Cell Biology and Genetics, Dresden, Germany). Rat anti-β1-integrin antibody (AIIB2, [46]) and mouse anti-α2-integrin (5E8, [47]) were kindly provided by Dr. K. Matlin (Department of Surgery, University of Chicago, Chicago, IL) and Dr. R.B. Bankert (Department of Microbiology and Immunology, University at Buffalo, Buffalo, NY), respectively. Mouse monoclonals anti-α3-integrin (VLA-3α), anti-β1-integrin (CD29) and anti-integrin-α6 (CD49f) were purchased from BD biosciences. Rabbit anti-cleaved caspase-3 was from Cell signaling technology. Rabbit anti-LNγ1 and mouse anti-γ-tubulin and anti-acetylated α-tubulin antibodies were from Sigma. Cy2-, Cy3- and HRP-conjugated secondary antibodies were all from Jackson Immunoresearch and Alexa488–conjugated secondary antibodies were from Invitrogen. TRITC-Phalloidin, DAPI, doxycycline and Y27632 were purchased from Sigma and Q-VD-OPh was from R&D Systems. Bovine dermal collagen I (PureCol™, Inamed), collagen IV (BD biosciences), BME (Matrigel™, BD biosciences) and placental laminin-511 (Sigma, L6274, [48]) were purchased. Retroviral constructs driving expression of GFP-Rac1^G12V^ (Addgene plasmid 14567) and GFP-Cdc42^Q61L^ (Addgene plasmid 14568) were obtained via Addgene [49].

### Cell culture and treatments

MDCK strain II cells (ATCC:CCL-34) and its derivative T23 MDCK-Rac1^T17N^ ([17], kindly provided by Dr. K. Matlin) were used in this study. For 2D-culture, the different MDCK cell lines were grown in minimum essential medium (MEM, Invitrogen) supplied with 5% fetal bovine serum (FBS, Perbio) and 1% penicillin/streptomycin at +37°C in a humidified CO_2_-incubator. 3D-cultures in gelatinous BME gels were prepared as described previously [50]. 2.5 mg/ml collagen gels were prepared as described previously [51]. When indicated, cells in 3D were cultured in the presence of 10 mM Q-VD-OPh, 5 µg/ml AIIB2, 20 ng/ml doxycycline or 10 µM Y27632. The growth medium was exchanged every 2–3 days.

### Adhesion and spreading assays

Adhesion assays were performed as described previously [10], [50]. Briefly, subconfluent cells were detached with 4 mM EDTA/1 mM EGTA in MEM. 1.5×10^5^ cells were washed and resuspended in MEM and pipetted into 96-well plates, which had been precoated with either laminin-511 (5 µg/ml), collagen I (9 µg/ml), BME (90 µg/ml) or collagen IV (10 µg/ml) and blocked with 1% Bovine serum albumin (BSA). Cells were allowed to adhere for 1,5 h after which non-adherent cells were removed by extensive washing with phosphate-buffered saline (PBS). Adherent cells were fixed with methanol and stained with crystal violet. Cells were lysed and absorbance was measured at 540 nm. For cell spreading analysis 1×10^4^ cells/cm^2^ in MEM containing 0.5% BSA were seeded onto ECM-coated glass coverslips. After 1.5 hours, cells were washed with PBS and fixed with 4% formaldehyde (PFA) followed by staining with TRITC-phalloidin (actin-staining to label the cell area) and DAPI (to label nuclei). A confocal image focusing at the cell-substrate interface was acquired and the average cell spread areas were determined using MacBiophotonics ImageJ software [52].

### Immunofluorescence and Immunoblotting

Immunofluorescence staining of cells grown in 2D and 3D cultures was done as described previously [50], [51]. For staining of extracellular laminin in live cells (Figure 3A), collagen cysts were incubated at +37°C with anti-laminin-γ1 mAb (diluted 1/100 in MEM) for 4 hours, after which gels were dissociated with 0.1% collagenase A (Roche) in MEM for 30 min at +37°C. Cysts were fixed with 4% PFA in PBS for 20 min, carefully washed and blocked with 0.5% BSA, 0.2% gelatin in PBS followed by addition of secondary antibodies. Samples were mounted on glass slides using Immumount (Shandon). For western immunoblotting of integrins, KD and control cells were seeded onto 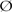 3.5 cm plastic tissue culture dishes and grown to confluency. Cells were lysed in TNE-buffer (10 mM Tris-HCl pH 7.4, 150 mM NaCl, 5 Mm EDTA, 2% Triton-X100) containing Complete Mini-protease inhibitor cocktail (Roche). Protein concentrations were determined with the BCA™ Protein Assay Kit (Pierce). Proteins were separate using SDS-PAGE (MiniProtean3 system, Biorad) and transferred onto Protran® nitrocellulose filters (Perkin-Elmer). Specific integrins bands were detected using 1/1000 dilutions of mouse monoclonal α3- and β1-integrin antibodies. Myc-epitope tagged Rac1-T17N was detected using 1/4000 dilution of the mouse monoclonal 9E10-antibody. HRP-conjugated secondary antibodies were visualized using a chemiluminescence kit (Pierce) and LAS-3000 imaging system (Fujifilm).

### Generation and modifications of KD -cells

Knockdown cells were created as described previously [53], [54]. Two functional constructs (>70% KD efficiency as determined by qPCR) were generated for each gene. For the target sequences see table S1. When indicated, individual integrin-KD shRNA-expressing MDCK cell clones were picked and expanded. These clonal cell lines were infected with retroviral constructs encoding for dominant active forms of Rac1 or Cdc42 or GFP.

### Time-lapse fluorescence microscopy

The cDNA of mouse E-cadherin was amplified using primers listed in Table S2 and ligated into a modified pN1-GFP vector using In-Fusion™ PCR cloning kit (Clontech). The resulting fusion protein expresses a full-length mouse E-cadherin followed by an in-frame linker sequence (GTVPGGGPGGGPGPVAT) and GFP before the stop codon. For live cell time-lapse movies of developing cysts, a stable MDCK cell line expressing mouse E-cadherin-GFP fusion protein was created and KDs were established as described above. E-cadherin-GFP-expressing KD and control cells were embedded into BME and imaged from day 1 until day 6 using an Olympus IX81 fluorescence microscope equipped with 40x UPLANFLN objective (NA:0.75), X-Cite 120 lightsource (EXFO Photonic Solutions Inc.) and an OKOLab Basic WJ CO_2_ Microscope Stage Incubator (OKOlab) adjusted to 37°C and 5% CO_2_. Pictures were collected with the XM10 CCD-camera (Olympus) at 20 minute intervals using Cell P -imaging software (GmbH). GFP-fluorescence was detected using 455–495 nm excitation filter and 510LP emission filter. Timelapse movies were assembled, cropped and contrasted using MacBiophotonics ImageJ software [52].

### Confocal microscopy

Confocal images were acquired at room temperature using an OLYMPUS FluoView-1000 laser-scanning confocal microscope (Olympus) and either 60x PlanSApo (NA:1.35) or 40x PlanApoF (NA:1.3) oil immersion objective. Sequential scans were performed using 405 nm, 488 nm and 543 nm laser lines for fluorophore excitation coupled with DAPI (430–470 nm), GFP (505–525 nm) and Cy3 (560LP) emission filters, respectively. Images were collected with the FV10-ASW software (Olympus) and imported into Adobe Photoshop CS (Adobe Systems, Inc.). For imaging of cysts, confocal stacks where collected with approximately 0.5 µm increments.

### Cdc42-activation assays

For analysis of Cdc42-activation, 3×10^5^ cells were seeded onto BME gel as described above. After 24 hours of culture, medium was replaced with serum-free MEM. After 48 hours of starvation, cells were lysed and Cdc42-activities were assayed according to manufacturers instructions (Cdc42 G-Lisa™, Cytoskeleton Inc.).

### Analysis of mitotic cells

KD and control cells grown 4 Days in BME gels were fixed and stained to visualize actin, acetylated α-tubulin and nuclei. Confocal stacks of the whole cyst structure were collected with approximately 0.5 µm increments. The X, Y and Z-coordinates of segregated post-metaphase chromatids and the centre of the cyst were measured with the MacBiophotonics ImageJ software. The relative distances in microns between the three points were calculated and the angles of the resulting triangle solved (See figure 5C). The distance from the midpoint between separated chromatins to the centre of the cyst was calculated and used for determination of the division angles.

### Statistics

Statistical analysis was carried out with the PASW statistics software (SPSS, Inc.). Normal distribution of samples was assessed with the Shapiro-Wilk test. Normally distributed samples were tested for significant differences by one-way ANOVA with multiple comparisons done by Tukeýs post hoc method for equal variances or Games-Howelĺs post hoc method for unequal variances. In some cases samples were presented as knockdown/control ratios and tested for significant differences from the value 1 with the Wilcoxon signed rank test or one sample t-tests when normally distributed. The distributions of measured cell division angles in KDs and controls were tested for significant differences using the Mann-Whitney-U test. P-values <0.05 were considered significant. P-values were not calculated for sample sizes smaller than 3.

## Supporting Information

Figure S1 **Itg-KDs efficiently reduce protein levels of abundantly expressed laminin- and collagen-binding integrins in MDCK cells.** A) MDCK cells were cultured on tissue-culture-treated Petri-dishes for 24 hours until they have reached 80% confluency. Total RNA was extracted, cDNA was synthesized and relative integrin mRNA levels were determined by qPCR using ubiquitin mRNA as an internal control. MDCK cysts were grown in 3D (2.5 mg/ml) collagen I gels for 6 days or in 3D BME gels for 3 days prior to RNA extraction and measurement of integrin mRNA levels as above. The length of cyst cultures prior to mRNA analysis was chosen to represent cultures where most of the cysts already show polarized morphology but some are still in a process of forming mature cysts. In all cases integrin mRNA levels are shown relative to expression to β1-integrin levels in 2D cultures. Data shows averages + SD from two independent measurements performed in duplicate. B) Lysates from puromycin-selected MDCK cells infected with control, Itgα3- or Itgβ1-KD viruses (upper panel) were assayed for expression of endogenous α3- and/or β1-integrin levels by western blotting. Two-fold and five-fold dilutions of the control cell lysates (1/1 = 20 µg of total protein) were loaded to allow residual integrin protein levels in the KD cells (20 µg of total protein loaded) to be estimated. α-tubulin was used as loading control. Due to antibody-related issues depletion of the protein levels of α2- and α6-integrins was assayed by immunofluorescence. Control, Itgα2- and Itgα6-KD MDCK cells were grown on Transwell-filters and stained for integrins as described in Experimental Procedures. Data is representative of two experiments with similar results. See also Table S1.
(TIF)

Figure S2 **Alternative shRNA constructs reproduce the observed knockdown phenotypes thereby confirming the specificity of the RNAi.** A) Control, Itgα3-, Itgα2-, Itgβ1- and Itgβ4-KD MDCK cells were allowed to settle for 90 minutes on collagen I (COL-I)-, basement membrane-extract (BME)-, laminin-511 (LN-511)- or collagen IV (COL-IV)-coated tissue culture wells. After washing, remaining adherent cells were fixed, stained and quantified. Absolute values were normalized to control values within the experiment (arbitrary units, AU). Data from 2–8 independent experiments per Itg-KD are shown with 25^th^, 50^th^ (median) and 75^th^ percentiles. P-values <0.05 are signified by (*), <0.01 by (**) and ≤0.001 by (***). B) Control, Itgα3-, Itgα2-, Itgβ1- and Itgβ4-KD MDCK cells were plated on COL-I or LN-511-coated glass coverslips. After 90 min of spreading, cells were fixed and filamentous actin stained using TRITC-Phalloidin. 37–250 cells from 11 pictures per sample were analyzed for spreading area. Average cell areas were normalized to controls (Arbitrary units, AU) within each experiment. Mean +SD of 2–5 independent experiments are shown. P-values <0.05 are signified by (*), <0.01 by (**) and P-values ≤0.001 by (***). C) Control, Itgα3-, Itgα2-, Itgβ1- and Itgβ4-KD MDCK cells were grown in 3D collagen I matrix for 10 days (Upper panel, COL-I) or in 3D BME gel for 7 days (lower panel, BME). Cysts were fixed and stained for DNA (DAPI, blue), filamentous actin (red) and an apical marker podocalyxin (green) as indicated. Cysts were phenotypically classified as normal when they had 1–2 central main apical lumen(s) with smooth contour. Cysts with poorly organized lumens, multiple small lumens or with no lumen were scored as abnormal. Size bars are 30 µm. D) Quantitation of the cyst phenotypes in collagen I matrix and BME. The data shows averages +SD from 2–5 independent experiments. A minimum of 150 cysts per sample was scored in each experiment. P-values <0.01 are signified by (**) and P-values ≤0.001 by (***). See also Figure 2.
(TIF)

Figure S3 **ECM-mediated orientation of the apico-basal axis depends on Rac1-activity in both collagen-I and BME gels.** T23 MDCK-Rac1^T17N^ cells were grown in 3D collagen I for 10 days or in BME gels for 7 days in the presence or absence of 20 ng/ml doxycycline. Cysts were fixed and stained for filamentous actin (TRITC-phalloidin, red), an apical marker (podocalyxin (Podxl), green) and nuclei (DAPI, blue). B) The cysts were phenotypically classified into three categories as described in Figure 2C. Data shows averages +SD from two independent experiments with duplicate samples. A minimum of 100 cysts were analyzed per sample. C) T23 MDCK-Rac1^T17N^ cells were grown on TC-treated plastic dishes for 48 hours in the presence or absence of 20 ng/ml doxycycline. Cells were lysed and expression of myc-tagged Rac1^T17N^ was analyzed by western blotting as described in materials and methods.
(TIF)

Figure S4 **Snapshots from the timelapse sequences of developing control and Itgα3-KD MDCK cysts.** Selected snapshots from timelapse series of E-cadherin-GFP-expressing control (movie S1) and Itgα3-KD (movie S2) MDCK cysts imaged at 20 minute intervals between day 1 to day 6 are shown. Yellow plate depicts the estimated position of the forming contractile ring.
(TIF)

Table S1 **shRNA target sequences used in the study and their respective mRNA depletion efficiencies.**
(DOC)

Table S2 **List of primers used for PCR.**
(DOC)

Movie S1 Cystogenesis of Ecad-GFP-expressing control cells in 3D BM gel (Days 1–6).
(MOV)

Movie S2 Cystogenesis of Ecad-GFP-expressing Itgα3-KD cells in 3D BM gel (Days 1–6).
(MOV)
