# Supplementary figures and images for: Two Distinct Integrin-Mediated Mechanisms Contribute to Apical Lumen Formation in Epithelial Cells
Source: PLoS One. 2011 May 6;6(5):e19453. doi: 10.1371/journal.pone.0019453 (PMC3089628; doi:10.1371/journal.pone.0019453)

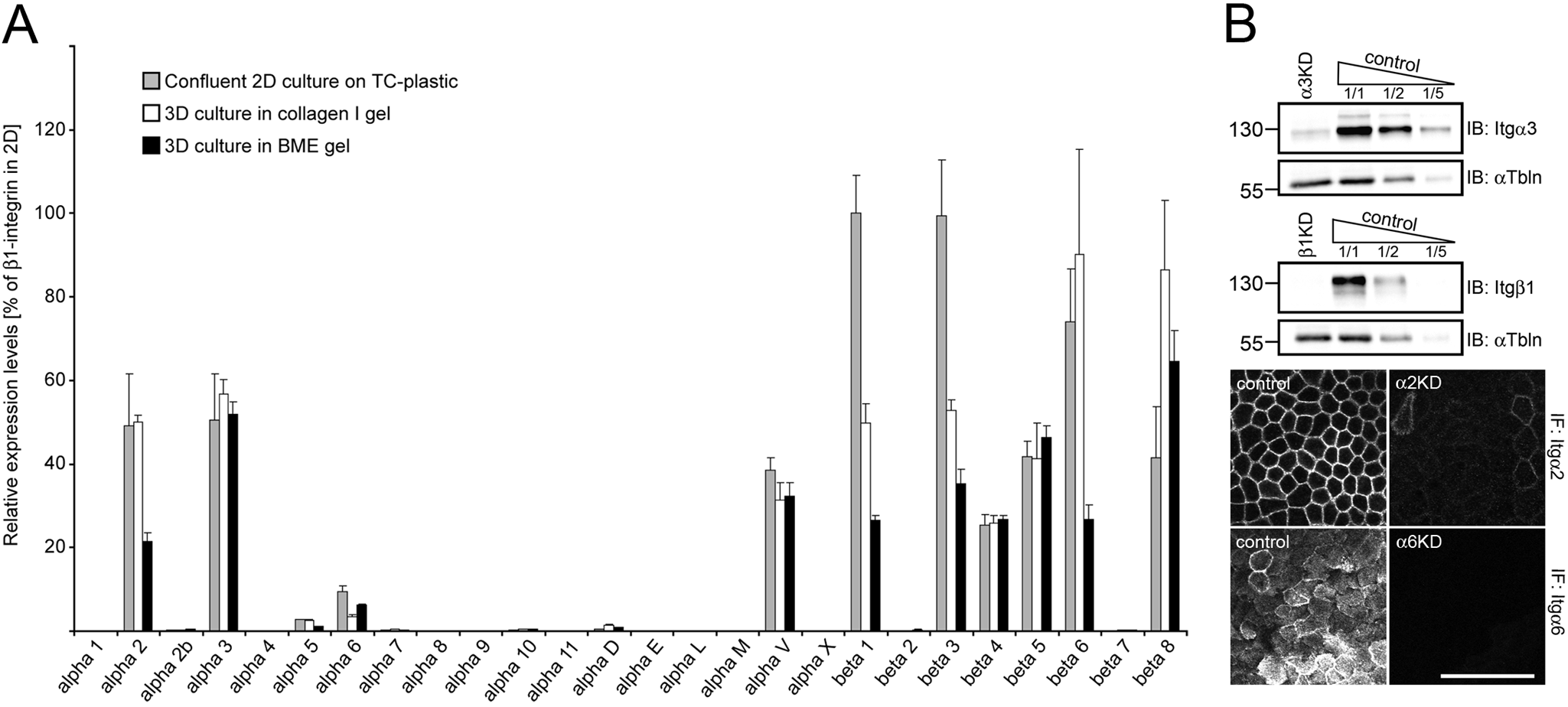

Supplement: Figure S1 — Itg-KDs efficiently reduce protein levels of abundantly expressed laminin- and collagen-binding integrins in MDCK cells. A) MDCK cells were cultured on tissue-culture-treated Petri-dishes for 24 hours until they have reached 80% confluency. Total RNA was extracted, cDNA was synthesized and relative integrin mRNA levels were determined by qPCR using ubiquitin mRNA as an internal control. MDCK cysts were grown in 3D (2.5 mg/ml) collagen I gels for 6 days or in 3D BME gels for 3 days prior to RNA extraction and measurement of integrin mRNA levels as above. The length of cyst cultures prior to mRNA analysis was chosen to represent cultures where most of the cysts already show polarized morphology but some are still in a process of forming mature cysts. In all cases integrin mRNA levels are shown relative to expression to β1-integrin levels in 2D cultures. Data shows averages + SD from two independent measurements performed in duplicate. B) Lysates from puromycin-selected MDCK cells infected with control, Itgα3- or Itgβ1-KD viruses (upper panel) were assayed for expression of endogenous α3- and/or β1-integrin levels by western blotting. Two-fold and five-fold dilutions of the control cell lysates (1/1 = 20 µg of total protein) were loaded to allow residual integrin protein levels in the KD cells (20 µg of total protein loaded) to be estimated. α-tubulin was used as loading control. Due to antibody-related issues depletion of the protein levels of α2- and α6-integrins was assayed by immunofluorescence. Control, Itgα2- and Itgα6-KD MDCK cells were grown on Transwell-filters and stained for integrins as described in Experimental Procedures. Data is representative of two experiments with similar results. See also Table S1. (TIF) [file pone.0019453.s001.tif]

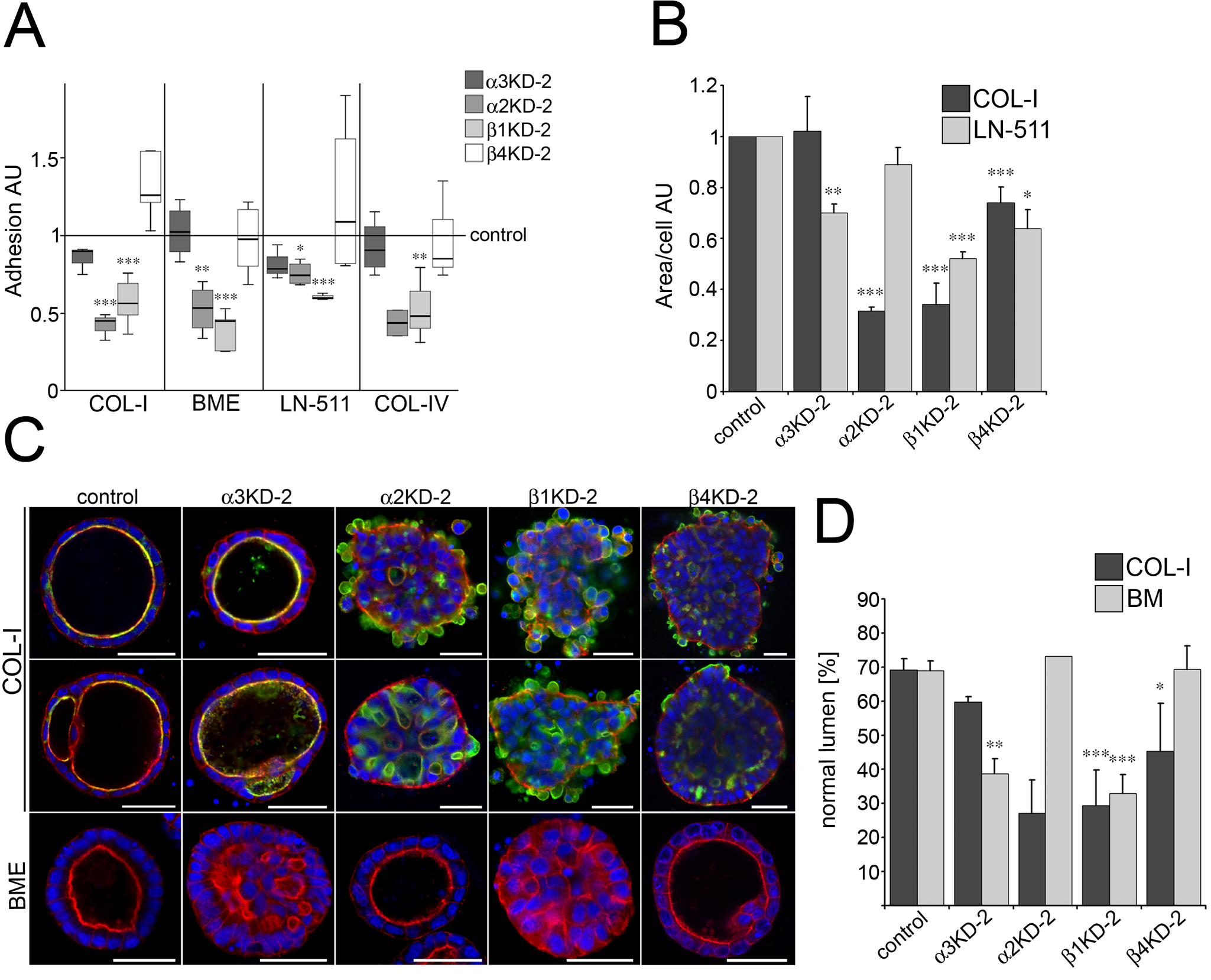

Supplement: Figure S2 — Alternative shRNA constructs reproduce the observed knockdown phenotypes thereby confirming the specificity of the RNAi. A) Control, Itgα3-, Itgα2-, Itgβ1- and Itgβ4-KD MDCK cells were allowed to settle for 90 minutes on collagen I (COL-I)-, basement membrane-extract (BME)-, laminin-511 (LN-511)- or collagen IV (COL-IV)-coated tissue culture wells. After washing, remaining adherent cells were fixed, stained and quantified. Absolute values were normalized to control values within the experiment (arbitrary units, AU). Data from 2–8 independent experiments per Itg-KD are shown with 25th, 50th (median) and 75th percentiles. P-values <0.05 are signified by (*), <0.01 by (**) and ≤0.001 by (***). B) Control, Itgα3-, Itgα2-, Itgβ1- and Itgβ4-KD MDCK cells were plated on COL-I or LN-511-coated glass coverslips. After 90 min of spreading, cells were fixed and filamentous actin stained using TRITC-Phalloidin. 37–250 cells from 11 pictures per sample were analyzed for spreading area. Average cell areas were normalized to controls (Arbitrary units, AU) within each experiment. Mean +SD of 2–5 independent experiments are shown. P-values <0.05 are signified by (*), <0.01 by (**) and P-values ≤0.001 by (***). C) Control, Itgα3-, Itgα2-, Itgβ1- and Itgβ4-KD MDCK cells were grown in 3D collagen I matrix for 10 days (Upper panel, COL-I) or in 3D BME gel for 7 days (lower panel, BME). Cysts were fixed and stained for DNA (DAPI, blue), filamentous actin (red) and an apical marker podocalyxin (green) as indicated. Cysts were phenotypically classified as normal when they had 1–2 central main apical lumen(s) with smooth contour. Cysts with poorly organized lumens, multiple small lumens or with no lumen were scored as abnormal. Size bars are 30 µm. D) Quantitation of the cyst phenotypes in collagen I matrix and BME. The data shows averages +SD from 2–5 independent experiments. A minimum of 150 cysts per sample was scored in each experiment. P-values <0.01 are signified by (**) and P-values [file pone.0019453.s002.tif]

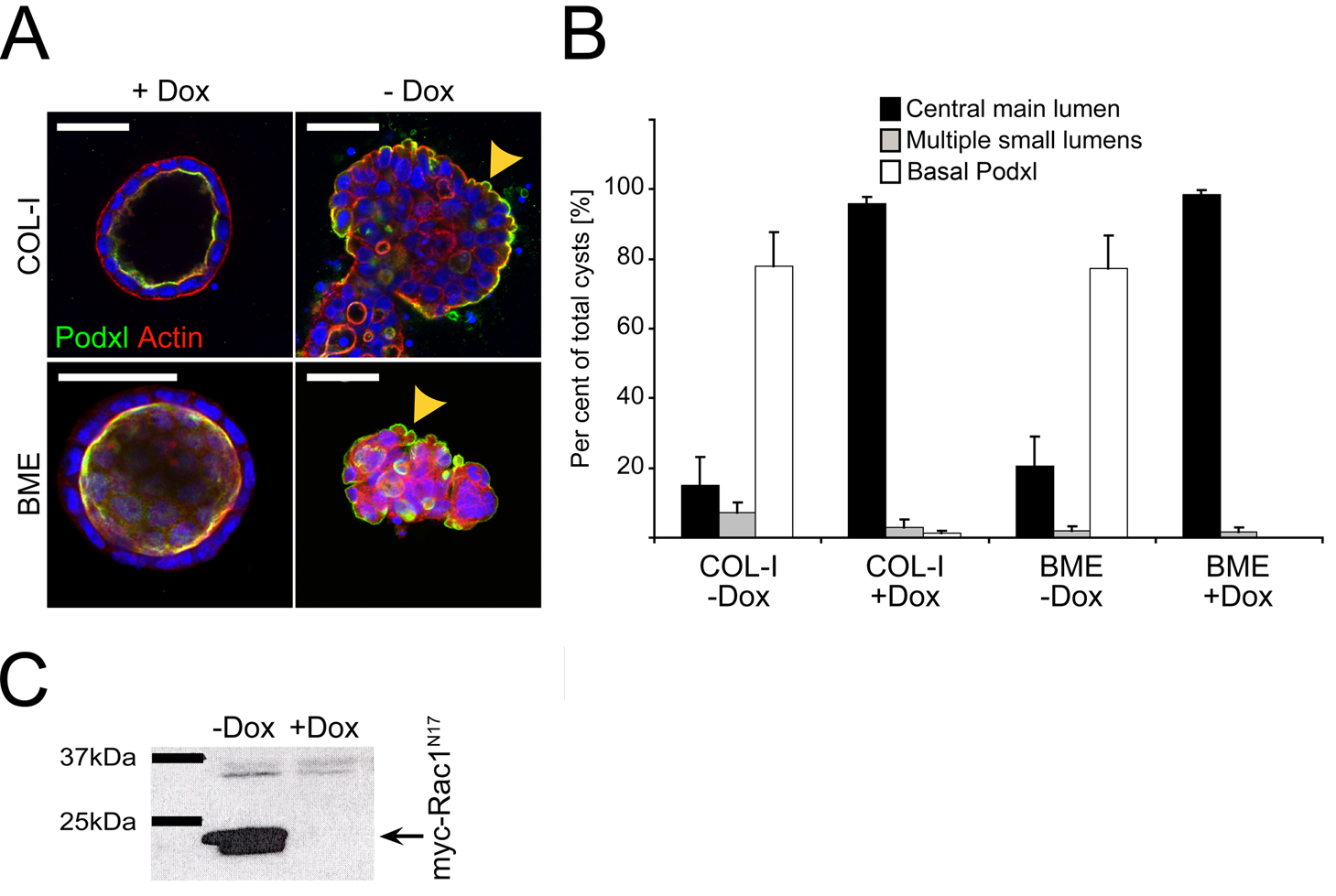

Supplement: Figure S3 — ECM-mediated orientation of the apico-basal axis depends on Rac1-activity in both collagen-I and BME gels. T23 MDCK-Rac1T17N cells were grown in 3D collagen I for 10 days or in BME gels for 7 days in the presence or absence of 20 ng/ml doxycycline. Cysts were fixed and stained for filamentous actin (TRITC-phalloidin, red), an apical marker (podocalyxin (Podxl), green) and nuclei (DAPI, blue). B) The cysts were phenotypically classified into three categories as described in Figure 2C. Data shows averages +SD from two independent experiments with duplicate samples. A minimum of 100 cysts were analyzed per sample. C) T23 MDCK-Rac1T17N cells were grown on TC-treated plastic dishes for 48 hours in the presence or absence of 20 ng/ml doxycycline. Cells were lysed and expression of myc-tagged Rac1T17N was analyzed by western blotting as described in materials and methods. (TIF) [file pone.0019453.s003.tif]

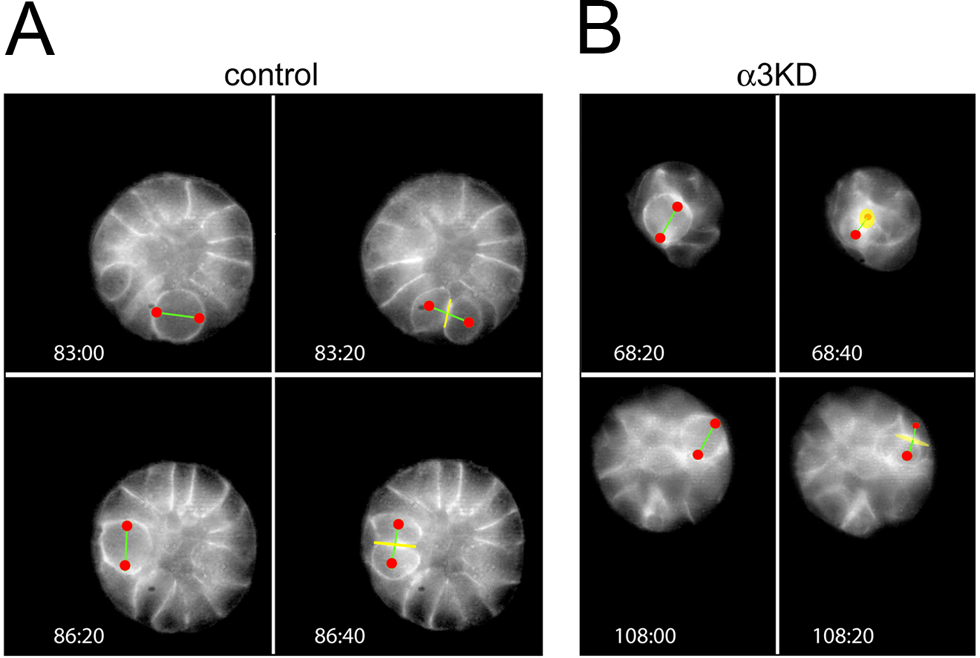

Supplement: Figure S4 — Snapshots from the timelapse sequences of developing control and Itgα3-KD MDCK cysts. Selected snapshots from timelapse series of E-cadherin-GFP-expressing control (movie S1) and Itgα3-KD (movie S2) MDCK cysts imaged at 20 minute intervals between day 1 to day 6 are shown. Yellow plate depicts the estimated position of the forming contractile ring. (TIF) [file pone.0019453.s004.tif]
