# Supplementary material for: Two Distinct Integrin-Mediated Mechanisms Contribute to Apical Lumen Formation in Epithelial Cells
Source: PLoS One. 2011 May 6;6(5):e19453. doi: 10.1371/journal.pone.0019453 (PMC3089628; doi:10.1371/journal.pone.0019453)
Supplement: Table S1 — shRNA target sequences used in the study and their respective mRNA depletion efficiencies. (DOC) [file pone.0019453.s005.doc]

**Table S1. shRNA target sequences used in the study and their respective mRNA depletion efficiencies**

| **Construct** | **Target sequence**^a^ | **%mRNA**^b^ | **Number of samples** |
| --- | --- | --- | --- |
| **α3KD-1** | GTGTAATAGCAACACTGACTA | 89.7±6.3 | 8 |
| **α3KD-2** | AGACCTACCACAATGAGATGT | 81,3±5.9 | 3 |
| **α6KD-1** | GTGACATGTGCTCACCGATAT | 83±10.2 | 7 |
| **α2KD-1** | GTGCAGATTACTCTCCTCAAA | 80.4±4 | 6 |
| **α2KD-2** | GGGAAAGTGCATACAACACTA | 79.2±4.7 | 6 |
| **β1KD-1** | AGGGACGTGTTGGTAGACATT | 90.2±3.9 | 5 |
| **β1KD-2** | AGTGCAGAGCCTTCAATAAAG | 72±5.1 | 5 |
| **β4KD-1** | GGAGAGCGGATTTCAGGCAAT | 68.5±0.3 | 2 |
| **β4KD-2** | GCCCTACAAGTTCAAGGTACA | 81.9±10.6 | 4 |

^a^ Target sequences were cloned into Retroviral RVH1-puro shRNA vectors as described previously [53,54]

^b^ Indicated as the percent decrease in levels of mRNA (relative to ubiquitin) comparing to control samples
