# Supplementary material for: Two Distinct Integrin-Mediated Mechanisms Contribute to Apical Lumen Formation in Epithelial Cells
Source: PLoS One. 2011 May 6;6(5):e19453. doi: 10.1371/journal.pone.0019453 (PMC3089628; doi:10.1371/journal.pone.0019453)
Supplement: Table S2 — List of primers used for PCR. (DOC) [file pone.0019453.s006.doc]

**Table S2. List of primers used for PCR**

| **Target** | **5´-Sense-3´** | **5´-Antisense-3´** |
| --- | --- | --- |
| **Itgα3** | CATCTACCACAGCAGCTCCA | CTCCTCCCCATGGATTACCT |
| **Itgα6** | AGTGGAGCTGTGGTTTTGCT | AGACCTTCCCCGTCAAAAAT |
| **Itgα2** | ATTTGGAAACTGCCACAAGC | CCAAGGCTCATGTTGGTTTT |
| **Itgβ1** | ATCCCAGAGGCTCCAAAGAT | GCTGGAGCTTCTCTGCTGTT |
| **Itgβ4** | ACAGTCCCAAGAAACGGATG | CCTTCACCGTGTAGCGGTAT |
| **Ubiquitin** | TCCAAGACAAGGAGGGCATC | TTCTAGCTGTTTGCCCGCA |
| **Ecad-GFP** | TACCGGACTCAGATCTCCGCC  ATGGGAG CCCGGTGCCGCA | CTGGACCTCCACCTGGTACCGTCCCGTCGTCCTCGCCACCGCCGT |
